# Juvenile Hormone-Receptor Complex Acts on *Mcm4* and *Mcm7* to Promote Polyploidy and Vitellogenesis in the Migratory Locust

**DOI:** 10.1371/journal.pgen.1004702

**Published:** 2014-10-23

**Authors:** Wei Guo, Zhongxia Wu, Jiasheng Song, Feng Jiang, Zhiming Wang, Shun Deng, Virginia K. Walker, Shutang Zhou

**Affiliations:** 1 State Key Laboratory of Integrated Management of Pest Insects and Rodents, Institute of Zoology, Chinese Academy of Sciences, Beijing, China; 2 School of Life Sciences, University of Science and Technology of China, Hefei, China; 3 Beijing Institutes of Life Science, Chinese Academy of Sciences, Beijing, China; 4 Department of Biology, Queen's University, Kingston, Ontario, Canada; University of Kentucky, United States of America

## Abstract

Juvenile hormone (JH), a sesquiterpenoid produced by the corpora allata, coordinates insect growth, metamorphosis, and reproduction. While JH action for the repression of larval metamorphosis has been well studied, the molecular basis of JH in promoting adult reproduction has not been fully elucidated. Methoprene-tolerant (Met), the JH receptor, has been recently shown to mediate JH action during metamorphosis as well as in vitellogenesis, but again, the precise mechanism underlying the latter has been lacking. We have now demonstrated using *Met* RNAi to phenocopy a JH-deprived condition in migratory locusts, that JH stimulates DNA replication and increases ploidy in preparation for vitellogenesis. *Mcm4* and *Mcm7*, two genes in the DNA replication pathway were expressed in the presence of JH and Met. Depletion of *Mcm4* or *Mcm7* inhibited *de novo* DNA synthesis and polyploidization, and resulted in the substantial reduction of *vitellogenin* mRNA levels as well as severely impaired oocyte maturation and ovarian growth. By using luciferase reporter and electrophoretic mobility shift assays, we have shown that Met directly regulates the transcription of *Mcm4* and *Mcm7* by binding to upstream consensus sequences with E-box or E-box-like motifs. Our work suggests that the JH-receptor complex acts on *Mcm4* and *Mcm7* to regulate DNA replication and polyploidy for vitellogenesis and oocyte maturation.

## Author Summary

Vitellogenesis, a hormonally-regulated process for the synthesis of yolk proteins by the fat body or liver and their sequestration in developing oocytes, takes place in all oviparous animals except mammals. Polyploidy has also been implicated in hormone-regulated development and reproduction. Although juvenile hormone (JH) is known to regulate polyploidy in insect models and also plays a pivotal role in stimulating insect vitellogenesis, the molecular mechanisms remain poorly understood. In the migratory locust, *Locusta migratoria*, vitellogenesis is dependent on JH, and JH stimulates DNA replication and increases ploidy in the fat body. Here, we show that a JH-receptor complex comprised of Methoprene-tolerant (Met) and a steroid receptor co-activator activates the transcription of two mini-chromosome maintenance (Mcm) genes, *Mcm4* and *Mcm7*. Knockdown of *Mcm4* or *Mcm7* via RNAi can phenocopy JH-deprivation and *Met*-depletion, resulting in reduced ploidy, blocked *vitellogenin* (*Vg*) expression, as well as arrested oocyte maturation and ovarian growth. This study provides evidence that JH acts through its receptor on the Mcm machinery to replicate the genome of fat body cells in preparation for the massive synthesis of Vg and possibly other proteins required for oocyte maturation and egg production.

## Introduction

Juvenile hormone (JH) is a key endocrine regulator controlling insect development, metamorphosis and reproduction. The larval characteristics of insects are maintained during 20-hydroxyecdysone (20E)-initiated molting in the presence of JH, whereas the absence of JH and a peak of 20E in final-instar larvae lead to metamorphosis. JH then reappears in adults to stimulate reproduction [1], [2]. Methoprene-tolerant (Met), a member of the basic helix–loop–helix (bHLH)-Per-Arnt-Sim (PAS) transcription factor family, has been recently identified as the JH receptor [3]–[5]. Met binds to JH with high affinity [5], [6]. Upon JH binding, Met forms a heterodimer with another bHLH-PAS transcription factor, the steroid receptor coactivator (SRC; also called Taiman in *Drosophila* or FISC in the mosquito, *Aedes aegypti*; we use SRC since the amino acid sequence of the locust orthologue shares a higher similarity to the SRC of the beetle, *Tribolium castaneum*). The Met-SRC heterodimer forms a ligand-dependent complex which can direct transcription of target genes in several insects [5], [7]–[9]. Met exerts its anti-metamorphic role by acting on *Krüppel homolog 1* (*Kr-h1*), which in turn represses the expression of *broad*, an early 20E response gene in metamorphosis [3], [7], [10].

Vitellogenesis is crucial to insect egg production and embryonic development. JH-dependent vitellogenesis has been reported in insects belonging to diverse orders, including Zygentoma, Orthoptera, Dictyoptera, Hemiptera, Coleoptera, Hymenoptera and Lepidoptera [1], [11], [12]. In *D. melanogaster*, both JH and 20E appear to be involved, although it is 20E that is responsible for high rates of vitellogenin (Vg) synthesis in the fat body [4], [13], [14]. In *Ae. aegypti*, JH plays an important priming role in preparing the fat body for vitellogenesis while 20E primarily controls Vg synthesis after a blood meal [15], [16]. In *T. castaneum*, JH regulates Vg synthesis in the fat body but 20E affects Vg synthesis through oocyte maturation [17]–[19]. Application of JH or a JH analog (JHA) induces *Vg* expression in the fat body of *T. castaneum*, whereas RNA interference (RNAi) targeting *Met* or a gene involved in JH synthesis (JH acid methyltransferase; JHAMT) results in the substantial reduction of *Vg* mRNA levels [18]. Knockdown of *Met* also blocks Vg synthesis and ovarian development in the linden bug, *Pyrrhocoris apterus* [20]. However, although JH has been known for decades to promote vitellogenesis in many insects, the molecular basis of this action is poorly understood, and the factors downstream of Met have not been well explored.

In adult females of the migratory locust, *Locusta migratoria*, JH acts independently of 20E to stimulate vitellogenesis and egg production [1], [11], [12]. JH induces Vg synthesis in locust fat body, and also initiates intercellular spaces, or patency, in the follicular epithelium to facilitate the uptake of Vg into developing oocytes [1], [16]. During vitellogenesis, locust fat body nuclei undergo extensive DNA replication to produce octaploid or more highly polyploid cells, which can be blocked by allatectomy and restored by topical application of a JHA [21], [22]. This JH-dependent polyploidization in the adult female is likely to facilitate an accelerated production of Vg and possibly other proteins required for reproduction.

In an effort to elucidate the mechanisms of JH action in locust vitellogensis, we employed an RNA-seq approach to identify differential gene expression (DGE) profiles in JH-deprived fat bodies and those further treated with a JHA. After JHA treatment, DNA replication was identified as a top ‘hit’ in the Kyoto Encyclopedia of Genes and Genomes (KEGG) pathways. Thus we postulated that Met mediated the JH-stimulated DNA replication in the fat body during locust vitellogenesis, and thus Met might directly regulate gene(s) involved in DNA replication. It is known that replicative helicase or the mini-chromosome maintenance (Mcm) proteins have essential roles in eukaryotic DNA replication [23]–[25]. Mcm4 is important for subunit interaction, licensing of DNA origins prior to S phase and the unwinding of DNA at replication forks [25]–[27]. Mcm7 contributes to the DNA helicase activity of the Mcm complex through interaction with other subunits [25]. We have shown here that *Mcm4* and *Mcm7* were expressed in response to JH, which in turn had a crucial role in polyploidy, Vg synthesis and oocyte maturation. *Met* RNAi mimicked the JH-deprived condition for DNA replication, polyploidy and vitellogenesis, and Met directly regulated the transcription of *Mcm4* and *Mcm7* by binding to identified upstream consensus sequences. Our present study thus provides insight into JH-dependent polyploidy and vitellogenesis.

## Results

### Juvenile hormone promotes DNA replication and polyploidy for vitellogenesis

**Figure 1:**
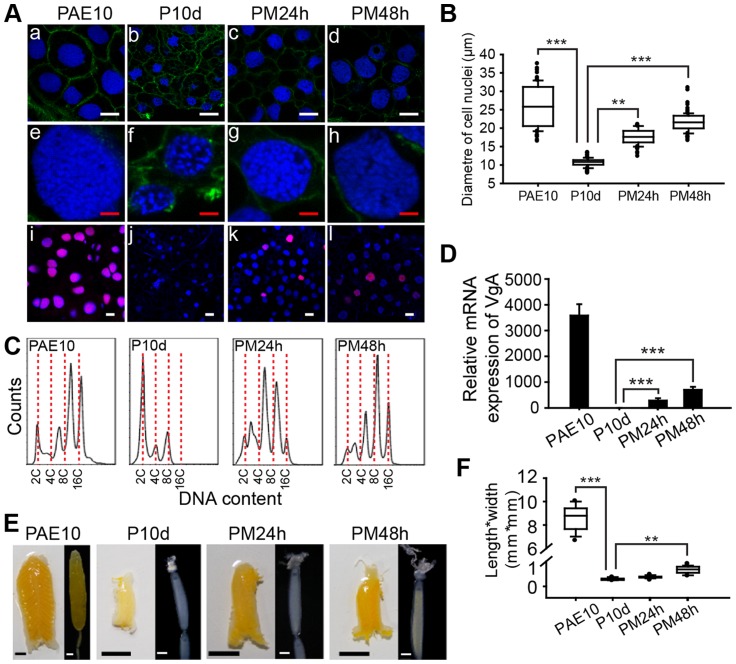
Effects of JH deprivation and JHA application on DNA replication and vitellogenesis. (A) Fat body cell ploidy (a–h) and DNA synthesis (i–l). PAE10, 10-day-old adult females; P10d, adult females treated with precocene for 10 d; PM24h and PM48h, precocene-treated adult females further treated with methoprene for 24 h and 48 h, respectively. Blue, nuclei; green, F-actin; red, *de novo* DNA synthesis. Enlarged images (4×) of a–d are shown in e–h, respectively. White bar, 20 µm; red bar, 5 µm. (B) Statistical analysis for the diameter of cell nuclei. **, *P*<0.01; ***, *P*<0.001; n = 80–100. (C) FACS analysis of DNA contents in fat body cells. (D) Relative mRNA levels of *VgA* in the fat body. ***, *P*<0.001; n = 8. (E) Morphology of ovaries and ovarioles. Scale bars: ovary, 5 mm; primary oocyte, 0.5 mm. (F) Statistical analysis for length*width index of primary oocytes. **, *P*<0.01; ***, *P*<0.001; n = 30.

Feulgen staining has shown that DNA synthesis and polyploidy in the adult female locust fat body are diminished after allatectomy and inducible by JHA application [22]. Here a JH-deprived condition was achieved by precocene treatment, with JH activity restored by methoprene. The detection of nuclei by staining with Hoechst-33342 and *de novo* DNA synthesis by the incorporation of 5-ethynyl-2′-deoxyuridine (EdU), followed by confocal microscopy, confirmed that chemical allatectomy inhibited *de novo* DNA synthesis (Fig. 1A). As a consequence there was substantially lower ploidy, and the mean fat body cell nuclear diameter (or the average of the major axis and minor axis for elliptical nuclei) decreased 2.6-fold in experimental females (Fig. 1A, B). Subsequent application of methoprene induced DNA synthesis and significantly increased ploidy (Fig. 1A). The mean nuclear diameter was increased 1.8- and 2.2-fold, respectively at 24 h and 48 h post methoprene treatment (Fig. 1B). Fluorescence-activated cell sorting (FACS) analysis revealed 8C and 16C peaks in female fat bodies at 10 days post adult eclosion (PAE), whereas precocene-treated fat bodies had dominantly 2C nuclei. Further methoprene treatment gave rise to 4C to 8C populations at 24 h, and chiefly 8C at 48 h (Fig. 1C).

Compared to that of control females, *VgA* (GenBank: KF171066) mRNA was detected at low levels in JH-deprived fat bodies but was dramatically induced 290-fold and 686-fold, respectively, by further methoprene treatment for 24 h and 48 h (Fig. 1D). It should be noted that the migratory locust has two *Vg* genes, *VgA* and *VgB*, which are coordinately induced by JH or JHA and expressed in similar patterns [28], [29], and thus *VgA* was selected as a representative. JH-deprived locusts showed arrested development of primary oocytes, ovarioles and ovaries, while further methoprene treatment stimulated their development (Fig. 1E) as evidenced by a 2.9-fold increase in the length*width index in primary oocytes (Fig. 1F). In contrast, acetone treatment (the solvent control) did not induce *VgA* expression, oocyte development or ovarian growth (Fig. S1). JH-deprived adult females had lower body weight compared to the controls, but there was no significant change in fat body size. Methoprene treatment for 24–48 h on JH-deprived locusts could not restore *VgA* mRNA, oocyte maturation and ovarian growth to levels seen in normal 10-day-old female adults (Fig. 1D–F). In our colonies, 10-day-old adult females showed a peak in *VgA* mRNA levels, and had fully developed ovaries with mature primary oocytes.

**Figure 2:**
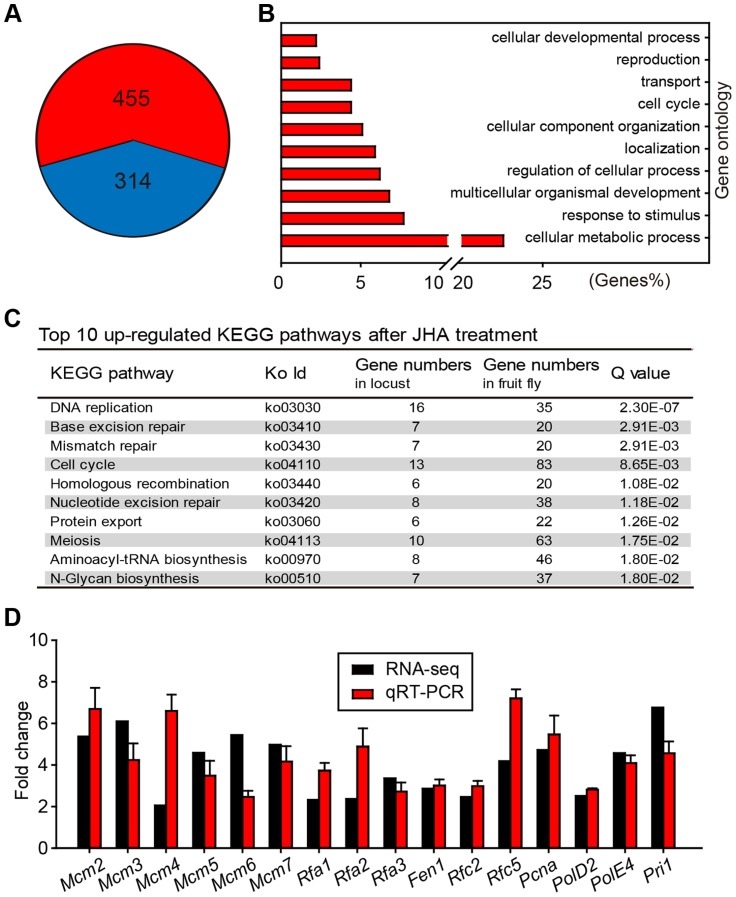
Differential gene expression profiles in JH-deprived and methoprene-exposed fat bodies. (A) Number of up-regulated (red) and down-regulated (blue) gene transcripts in JH-deprived fat bodies further treated with methoprene for 24 h. Fold change ≥2 and *P*<0.05 were used as the cutoff criteria. (B) Significantly enriched gene ontology terms associated with up-regulated genes. (C) Significantly enriched KEGG pathways (top 10) of up-regulated genes. (D) Validation of RNA-seq data by qRT-PCR for genes associated with DNA replication. Fold change was calculated as: mRNA levels in methoprene-treated females/mRNA levels in precocene-treated females.

Using RNA-seq analysis, we identified 455 up-regulated and 314 down-regulated genes in JH-deprived fat bodies further treated with methoprene for 24 h (Fig. 2A, Table S1). Gene ontology analysis showed that the up-regulated genes were significantly enriched in cellular metabolic processes, response to stimuli, development, and cellular process regulation (Fig. 2B). KEGG pathway analysis showed that DNA replication was on the top of up-regulated pathways (Fig. 2C). Subsequent real-time semi-quantitative reverse transcription PCR (qRT-PCR) confirmed that the expression of all 16 genes in the DNA replication pathway were up-regulated in methoprene-treated (Fig. 2D), but not acetone-treated (Fig. S2) fat bodies when compared to the precocene treatment.

### *Met* RNAi phenocopies JH deprivation in DNA replication and polyploidy

**Figure 3:**
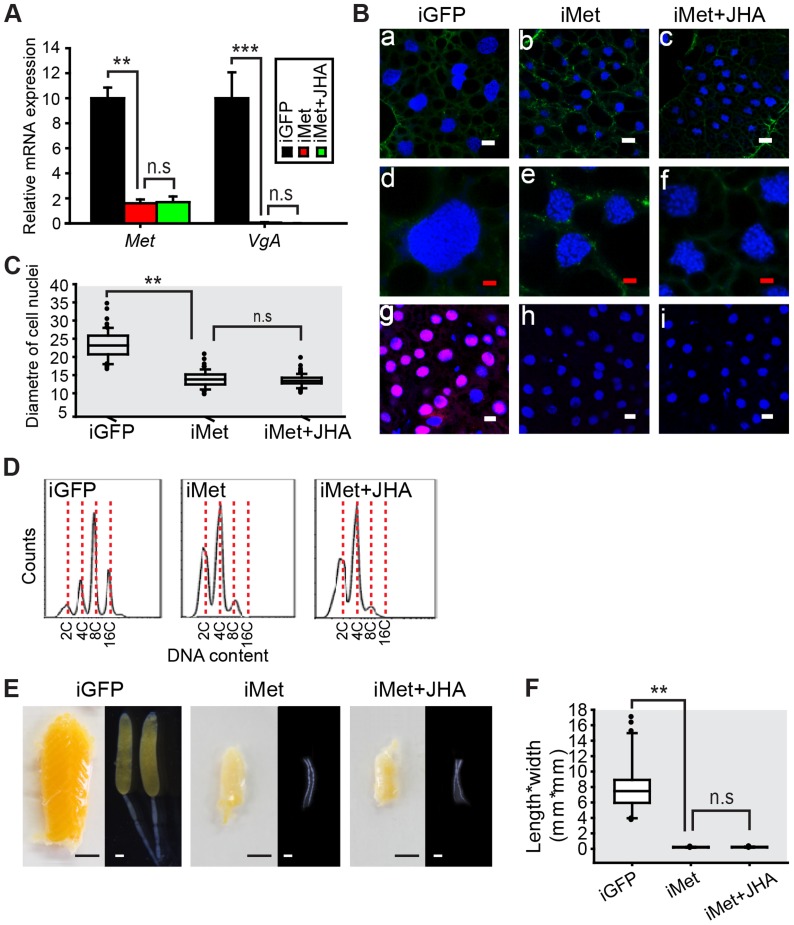
Effects of *Met* RNAi on DNA replication and vitellogenesis. (A) *Met* knockdown efficiency and *VgA* mRNA levels in the fat body of the dsGFP control (iGFP), *Met* RNAi (iMet), and iMet further treated with methoprene for 48 h (iMet+JHA). **, *P*<0.01 and ***, *P*<0.001; n.s, no significant difference; n = 4–6. (B) Fat body cell ploidy (a–f) and DNA synthesis (g–i) of iGFP, iMet and iMet+JHA. Blue, nuclei; green, F-actin; red, *de novo* DNA synthesis. Enlarged images (4×) of a–c are shown in d–f, respectively with the white bar, 20 µm and red bar, 5 µm. (C) Statistical analysis for the diameter of cell nuclei. **, *P*<0.001; n = 80–100. (D) FACS analysis of DNA contents in fat body cells. (E) Representative phenotypes of ovaries and ovarioles after iMet, iMet+JHA *vs.* iGFP. (F) Statistical analysis for length*width index of primary oocytes. ***, *P*<0.001; n = 30.

The sequenced *L. migratoria* genome [30] and transcriptome [31] yielded a single *Met* gene without isoforms. We cloned locust *Met* (GenBank: KF471131) cDNA by RACE PCR and performed RNAi in order to evaluate the role of Met in JH-dependent polyploidy and vitellogenesis. During the first gonadotrophic cycle of female adults, vitellogenesis started ∼5 days post eclosion under our rearing conditions. Thus we used 6-day-old adult females for the evaluation of gene knockdown efficiency and *Vg* expression. Injection of *Met* dsRNA reduced *Met* mRNA to 16% of its normal levels in the fat body, compared to the *GFP* dsRNA (iGFP) control (Fig. 3A). Significantly, this *Met* RNAi (iMet) resulted in a 99.9% reduction of *VgA* mRNA levels (Fig. 3A), as well as considerably lower fat body ploidy, and no detectable *de novo* DNA synthesis (Fig. 3B). The diameter of *Met*-depleted fat body cell nuclei was ∼58% of iGFP controls (Fig. 3C). Distinct from the 8C peak as well as the 4C and 16C populations in the fat body of iGFP controls, only 2C and 4C peaks were observed after iMet treatment (Fig. 3D). Further application of methoprene on iMet locusts did not rescue the reduced *VgA* expression, DNA synthesis or DNA content (Fig. 3A–D). During vitellogenesis, primary oocytes of iGFP controls grew exponentially with the uptake of yolk proteins. Consequently, the iGFP control ovaries became big and yellow (Fig. 3E). In contrast, the primary oocytes of iMet or methoprene-exposed iMet females did not grow and the ovaries remained small and white (Fig. 3E). The length*width index of primary oocytes significantly decreased 34.7-fold after iMett, and further methoprene treatment failed to restore oocyte growth (Fig. 3F). The survival rates in iGFP, iMet and iMet+methoprene treatment groups were 92%, 80% and 84%, respectively. The described phenotypes were consistently observed in 95% of surviving iMet females. Notably, the capacity of methoprene to induce DNA replication and *VgA* expression was completely blocked by *Met* RNAi.

### *Mcm4* and *Mcm7* are expressed in the presence of JH and Met

**Figure 4:**
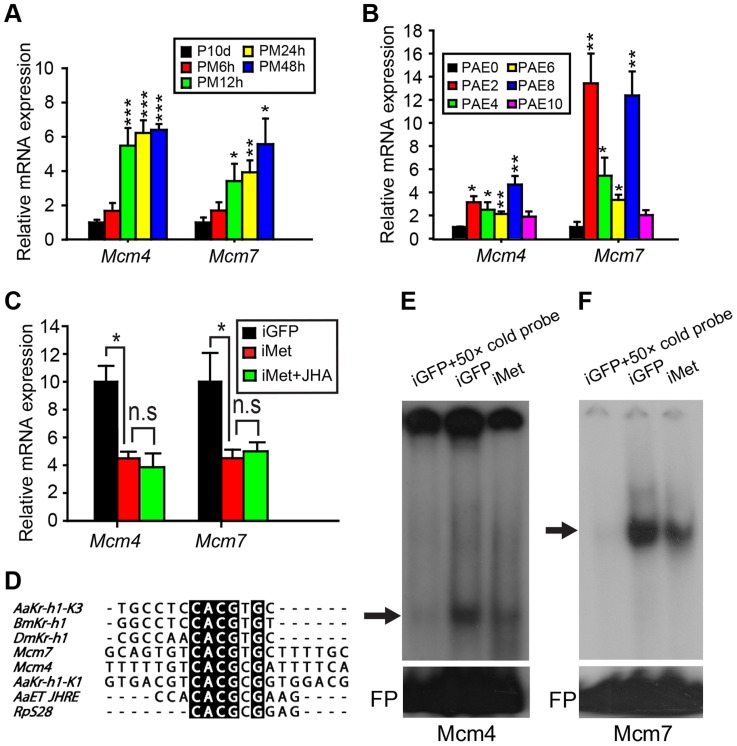
Responsiveness of *Mcm4* and *Mcm7* to JH and *Met* RNAi. (A) Relative mRNA levels of *Mcm4* and *Mcm7* in the fat body of adult females treated with precocene (P10d) and those further treated with methoprene for 6, 12, 24 and 48 h (PM6h, PM12h, PM24h and PM48h, respectively). mRNA levels in precocene-treated fat bodies were used as the calibrator. *, *P*<0.05, **, *P*<0.01 and ***, *P*<0.001 compared to P10d; n = 4–6. (B) *Mcm4* and *Mcm7* mRNA abundance in fat bodies of female locusts from 0 to 10 days post adult eclosion (PAE). *, *P*<0.05 and **, *P*<0.01 compared to PAE0; n = 4–6. (C) Relative levels of *Mcm4* and *Mcm7* transcripts in fat bodies of dsGFP control (iGFP), *Met*-RNAi (iMet), and iMet further treated with methoprene for 48 h (iMet+JHA). *, *P*<0.05; n = 10–12. (D) Alignment of DNA element sequences containing E-box and E-box-like motifs in the upstream promoter regions of locust *Mcm7* and *Mcm4* with experimentally tested Met-binding consensus sequences except that of *DmKr-h1* [7], [9], [34]–[36]. (E) EMSA using the Mcm4 probe listed in (D) with fat body nuclear extracts of iGFP and iMet (shown is a representative short exposure; longer exposures in some experiments showed additional two bands of less interest; see Results and Fig. S7). (F) EMSA using Mcm7 probe listed in (D) and with fat body nuclear extracts of iGFP and iMet. For both (E) and (F), arrows indicate the most likely specific bands. FP, free probe.

After RNA-seq-based gene expression profiling and subsequent qRT-PCR validation, 16 genes associated with DNA replication were identified as up-regulated in methoprene-treated fat bodies (Fig. 2C and D; Table S1). Of these, six *Mcm* sequences were subsequently analyzed in more detail since they are known to be critical for DNA replication [23], [25], [32]. To reveal the dynamics of JH-stimulated transcription, *Mcm2-7* (GenBank: KM036511, KM036512, KF471133, KM036513, KM036514 and KF471134, respectively) mRNA levels were further assessed by qRT-PCR at additional times after methoprene treatment, using total RNA from JH-deprived fat bodies as well as those further treated with methoprene for 6, 12, 24 and 48 h. Compared to JH-deprived fat bodies, *Mcm2-7* mRNA levels were significantly increased 2.6- to 5.5-fold in fat bodies after methoprene treatment at 12 h and remained high at 24–48 h (Fig. 4A, Fig. S3). In parallel experiments, acetone treatments (6–48 h) had no significant effect on monitored mRNA levels (Fig. S4). To explore the temporal abundance of *Mcm2-7* mRNAs during vitellogenesis, qRT-PCR was carried out using total RNA from adult female fat bodies collected at 0, 2, 4, 6, 8 and 10 days PAE. Despite overall fluctuations in mRNA levels, *Mcm2-7* mRNA levels were significantly higher at 2 and 8 days PAE compared to the day of eclosion (0 day PAE) (Fig. 4B, Fig. S5).

Interestingly, significant increases in mRNA levels from 2 to 8 days PAE were observed for *Mcm4* and *Mcm7* (Fig. 4B). *Mcm4* mRNA levels increased 3.1-fold on day 2, remained high on day 4–6, and reached a peak on day 8 (Fig. 4B). Thereafter (day 10), *Mcm4* mRNA decreased to levels that were similar to those seen on day 0 (Fig. 4B). Somewhat similar in pattern, *Mcm7* mRNA levels increased 13.4-fold on day 2, remained elevated on day 4 and 6 (5.4- and 3.3- fold, respectively), and then increased again (12.4-fold) on day 8, before declining to levels seen on day 0 (Fig. 4B). We next investigated the effects of *Met* RNAi on *Mcm2-7* expression. Fat bodies from iMet-treated females showed significant reductions of 74.6%, 57.5% and 55.8% in *Mcm3*, *Mcm4* and *Mcm7* mRNA levels, respectively (Fig. 4C, Fig. S6). However, *Met* knockdown had no significant impact on the expression of *Mcm2*, *Mcm5* or *Mcm6* (Fig. S6). Further methoprene treatment on iMet locusts did not rescue the reduced expression of *Mcm4* or *Mcm7* (Fig. 4C), indicating the essential role of Met in JH-stimulated *Mcm4* and *Mcm7* expression.

### *Mcm4* and *Mcm7* are regulated by the JH-receptor complex

Analysis of upstream regions of *Mcm4* and *Mcm7* (3 kb each) revealed a palindromic canonical E-box, CACGTG, in the proximal promoter region (nt −662 to −657) of *Mcm7* and an E-box-like motif, CACGCG, in the promoter region (nt −359 to −354) of *Mcm4* (Fig. 4D). An E-box is a characteristic signature of recognition for bHLH-PAS transcription factors [33], and E-box or E-box-like motifs have been previously reported for Met binding [7], [9], [34]–[36]. Therefore, electrophoretic mobility shift assays (EMSA) were conducted using nuclear extracts from *Met*-depleted *vs.* dsGFP-treated fat bodies with 20-mer nucleotide probes (Fig. 4D) corresponding to the sequences containing the conserved E-box or E-box-like motifs. When short exposure times of the EMSA films were used, a single band was visualized with the ^32^P-Mcm4 probe, which was abolished by competition with 50× molar excess of unlabeled Mcm4 probe. The band's intensity was reduced when nuclear extracts from *Met*-depleted fat bodies were incubated with the ^32^P-Mcm4 probe (Fig. 4E). It must be noted that after longer exposure times in some experiments, additional two bands were visualized (Fig. S7). The fastest moving band (corresponding to the single band described above) was similarly eliminated with 50× molar excess of unlabeled Mcm4 probe. The middle band was reduced, but not abolished with unlabeled probe, and was unaffected by *Met*-depleted fat body nuclear extracts; there was no competition-mediated reduction in intensity with the slowest moving band (Fig. S7). After using the ^32^P-Mcm7 probe in EMSA, a strong band with lower mobility was observed with nuclear extracts derived from dsGFP-treated fat bodies. This band was diminished with 50× molar excess of unlabeled Mcm7 probe, and also reduced in intensity when *Met*-depleted fat body nuclear extracts were used (Fig. 4F). These data suggest the possible involvement of Met in the specific complexes with sequences represented by the Mcm4 and Mcm7 probes. Although, as indicated, *Mcm3* also showed a significant reduction in mRNA levels after *Met* RNAi, we were unable to determine the presence of E-box or E-box-like motifs in the promoter of *Mcm3* due to the unavailability of the upstream sequence and thus EMSA analysis was not possible.

**Figure 5:**
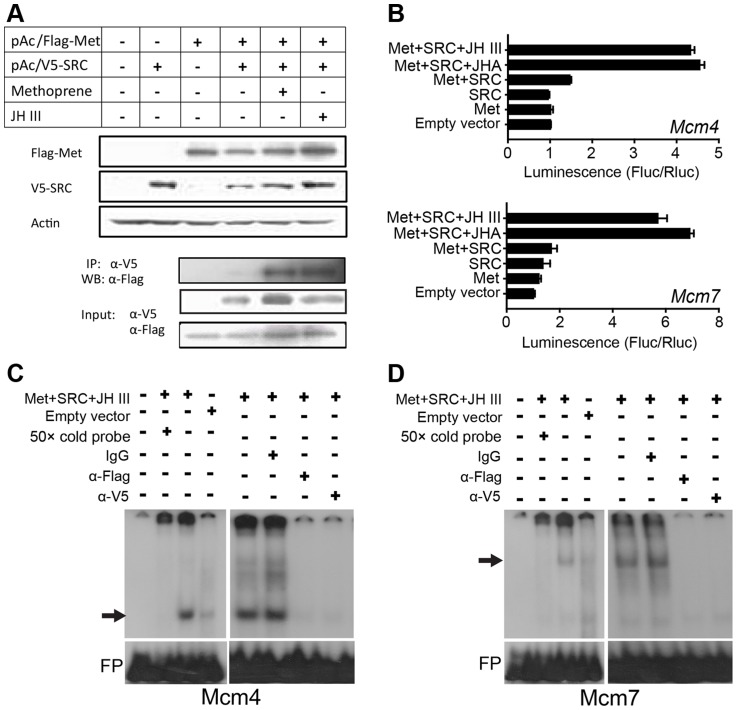
*Mcm4* and *Mcm7* transcription and the JH-receptor complex. (A) Upper panels: western blot (WB) showing the expression of Flag-Met^1-3108^ and V5-SRC in S2 cells; lower panel: immunoprecipitation (IP) showing the interaction of Flag-Met^1-3108^ and V5-SRC in the presence of 10 µM JH III or methoprene. α-Flag, Flag antibody; α-V5, V5 antibody. (B) Luciferase assays after co-transfection of pGL4.10/*Mcm4* ^−933 to −37^ or pGL4.10/*Mcm7* ^−933 to −11^ into S2 cells compared with the pAc5.1 empty vector, pAc5.1/Flag-Met^1-3108^ (Met) or/and pAc5.1/V5-SRC (SRC), with or without 10 µM JH III or methoprene (JHA) treatment. (C) EMSA using the Mcm4 probe and S2 cell nuclear extracts with expressed Flag-Met and V5-SRC and treated with JH III (10 µM). The arrow indicates the specific complex. FP, free probe. (D) EMSA using the Mcm7 probe and S2 cell nuclear extracts with expressed Flag-Met and V5-SRC and treated with JH III (10 µM). The arrow indicates the specific complex. FP, free probe.

To determine if Met directly regulates *Mcm4* and *Mcm7* transcription, *Met* cDNA (nt 1–3108) was cloned into a pAc5.1/Flag vector to express Flag-Met^1-1036^ fusion proteins in *Drosophila* S2 cells. Since SRC/FISC/Taiman functions as the heterodimeric partner of Met [3], [5], [8], [9], we also cloned its locust ortholog (GenBank: KF471132) into a pAc5.1/V5 vector to obtain V5-SRC fusion proteins. Western blot and immunoprecipitation demonstrated that in the presence of JH III or methoprene, recombinant Flag-Met and V5-SRC were associated as heterodimers (Fig. 5A). This result suggests that JH is necessary for Met-SRC interaction.

In luciferase reporter assays, the upstream regulatory sequences of *Mcm4* (nt −933 to −37) or *Mcm7* (nt −933 to −11) was cloned into the pGL4.10 vector. The constructs were then co-transfected along with pAc5.1/Flag-Met, pAc5.1/V5-SRC, or pAc5.1/Flag-Met plus pAc5.1/V5-SRC into *Drosophila* S2 cells. The pAc5.1/V5 empty vector was used as the control. To minimize the possible interference of endogenous Met, Gce (Germ cell-expressed) and Taiman, these three genes were knocked down in S2 host cell lines via treatment with *Drosophila Met* and *Taiman*-specific dsRNA (the sequence of *Met* dsRNA shares about 40% identity to that of *Gce*). These treatments reduced *Drosophila Met*, *Gce* and *Taiman* transcript abundance to 28.4%, 48.6% and 10.2%, respectively, of their normal levels (Fig. S8). In contrast, after transfection, *Flag-Met* and *V5-SRC* mRNA levels showed no significant change (Fig. S8). As shown in Fig. 5B, without JH III or methoprene treatment, the co-expression of Flag-Met and V5-SRC was unable to induce *Mcm4* or *Mcm7* reporter activity, similar to the expression of Flag-Met or V5-SRC alone. However, in the presence of JH III or methoprene, the co-expression of Flag-Met and V5-SRC led to the 4.3–6.9 fold increase in luciferase activities compared to the control (Fig. 5B). This indicates that JH-induced Met-SRC interaction is required for *Mcm4* and *Mcm7* transcription.

To further confirm Met binding to the *Mcm4* and *Mcm7* promoters, we performed EMSA using nuclear extracts from S2 cells co-transfected with pAc5.1/Flag-Met and pAc5.1/V5-SRC followed by JH III treatment. As shown in Fig. 5C, a specific band was seen with the ^32^P-Mcm4 probe, but it disappeared after 50× molar excess of the unlabeled Mcm4 probe was added to the reaction. Pre-incubation of the nuclear extracts with the anti-Flag or anti-V5 antibody resulted in the loss of this band, whereas pre-incubation with IgG had no effect on its mobility or intensity (Fig. 5C). A specific band with a slower mobility was observed with the ^32^P-Mcm7 probe, which was similarly eliminated by the addition of 50× molar excess of unlabeled Mcm7 probe in the binding reaction (Fig. 5D). This band also disappeared when the nuclear extracts were pre-incubated with anti-Flag or anti-V5 antibody, but not IgG (Fig. 5D). Together these observations indicate that the locust Met has specificity for *Mcm7* E-box and *Mcm4* E-box-like motifs. Fainter, but still specific bands with the empty vector were seen for both Mcm4 and Mcm7 probes, which could imply possible weak binding by endogenous Met/Gce and Taiman in S2 cells [37], [38].

### *Mcm4* or *Mcm7* knockdown blocks DNA replication, polyploidy and vitellogenesis

**Figure 6:**
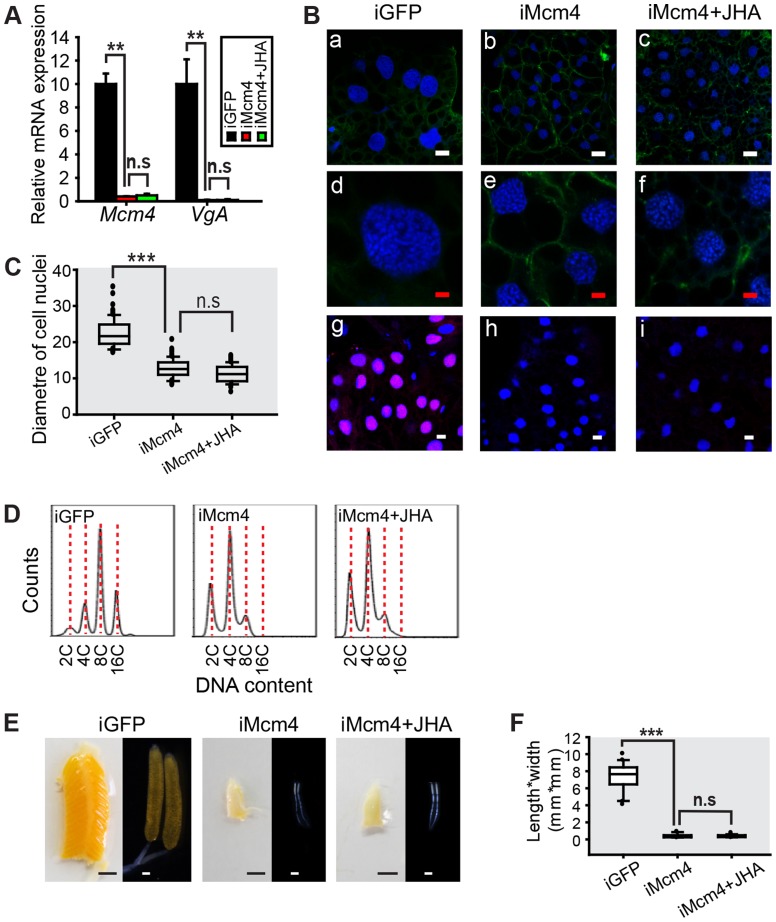
Effect of *Mcm4* RNAi on DNA replication, ployploidy and vitellogenesis. (A) *Mcm4* knockdown efficiency and *VgA* mRNA levels in the fat body of the dsGFP control (iGFP), *Mcm4* RNAi (iMcm4), and iMcm4 further treated with methoprene for 48 h (iMcm4+JHA). **, *P*<0.01; n.s, no significant difference; n = 4–6. (B) Fat body cell ploidy (a–f) and DNA synthesis (g–i) of iMcm4, iMcm4+JHA compared to iGFP. Blue, nuclei; green, F-actin; red, *de novo* DNA synthesis. Enlarged images (4×) of a–c are shown in d–f, respectively. White bar, 20 µm; red bar, 5 µm. (C) Statistical analysis for the diameter of cell nuclei. ***, *P*<0.001; n = 80–100. (D) FACS analysis of DNA contents in fat body cells after iMcm4 and iMcm4+JHA *vs.* iGFP. (E) Representative phenotypes of ovaries and primary oocytes after iMcm4 and iMcm4+JHA. (F) Statistical analysis for length*width index of primary oocytes. ***, *P*<0.001; n = 30.

*Mcm4* RNAi (iMcm4) was highly efficient since fat body *Mcm4* mRNA levels were reduced to 4% of iGFP controls. Under these conditions, *VgA* mRNA levels were also reduced to about 0.1% of iGFP controls (Fig. 6A). Methoprene treatment on the *Mcm4*-depleted locusts failed to increase *VgA* mRNA levels (Fig. 6A), indicating that the loss of *Mcm4* function precluded methoprene-induced *VgA* expression. Similar to JH-deprivation and *Met*-depletion groups, *Mcm4*-depleted fat bodies showed blocked *de novo* DNA synthesis and lower ploidy (Fig. 6B). Mean nuclear diameters were decreased about 43% in iMcm4 fat bodies compared to iGFP controls (Fig. 6C). Flow cytometry supported the cytological analysis and showed that *Mcm4*-depleted fat bodies had a lower DNA content, with a 2C population and a peak at 4C, compared to iGFP controls with 4C and 16C populations and a peak at 8C (Fig. 6D). Strikingly, compared to a 93% survival rate in iGFP controls, 37% of iMcm4 females did not survive to day 8 PAE, indicating the importance of Mcm4 in adult development. Of those that did survive, 95% of iMcm4 females had severely impaired oocyte maturation and ovarian growth (Fig. 6E), underscored by a dramatically lower length*width index of primary oocytes (Fig. 6F). Additional application of methoprene on iMcm4 locusts did not rescue the *Mcm4*-depleted phenotype (Fig. 6B–F).

**Figure 7:**
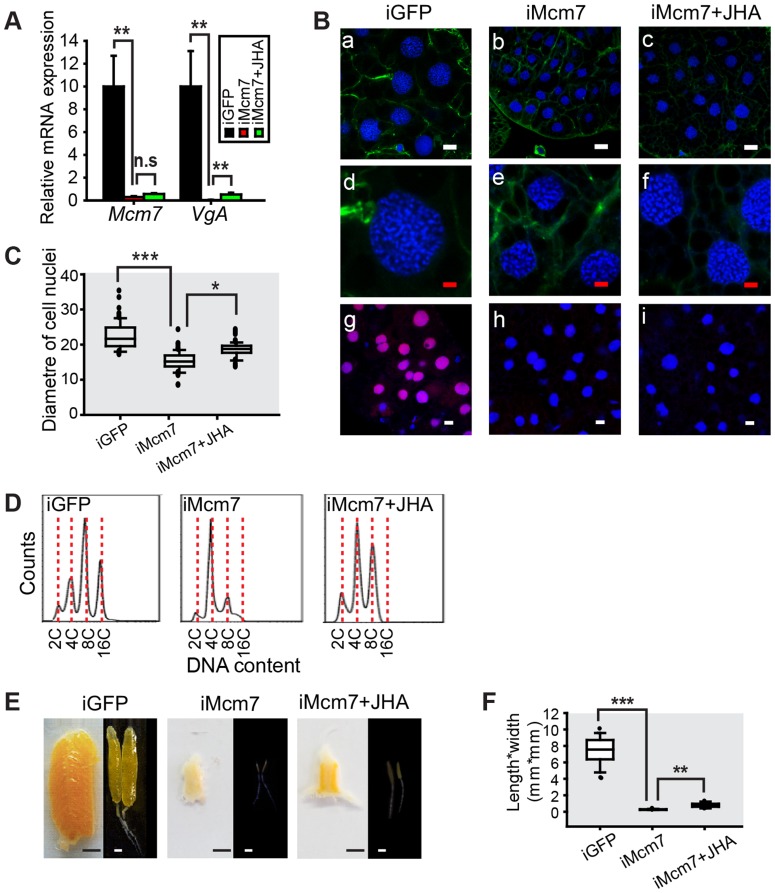
Effect of *Mcm7* RNAi on DNA replication, ployploidy and vitellogenesis. (A) *Mcm7* knockdown efficiency and *VgA* mRNA levels in the fat body of dsGFP control (iGFP), *Mcm7* RNAi (iMcm7), and iMcm7 further treated with methoprene for 48 h (iMcm7+JHA). **, *P*<0.01; n.s, no significant difference; n = 4–6. (B) Comparison of fat body cell ploidy (a–f) and DNA synthesis (g–i) among iGFP, iMcm7 and iMcm7+JHA groups. Blue, nuclei; green, F-actin; red, *de novo* DNA synthesis. Enlarged images (4×) of a–c are shown in d–f, respectively. White bar, 20 µm; red bar, 5 µm. (C) Statistical analysis for the diameter of cell nuclei. *, *P*<0.05; ***, *P*<0.001; n = 80–100. (D) FACS analysis of DNA content in fat body cells after iMcm7 and iMcm7+JHA compared to iGFP. (E) Representative phenotypes of ovaries and primary oocytes after iMcm7 and iMcm7+JHA. (F) Statistical analysis for length*width index of primary oocytes. **, *P*<0.01; ***, *P*<0.001; n = 30.

In the fat body of *Mcm7* RNAi (iMcm7) females, there was a 97.1% reduction in *Mcm7* mRNA levels and *VgA* transcript abundance declined by 99.97% (Fig. 7A). *Mcm7* knockdown also inhibited *de novo* DNA synthesis, resulting in lower ploidy (Fig. 7B). The nuclear diameter in iMcm7 fat body cells was ∼67% of the iGFP controls (Fig. 7C), and cells were chiefly 4C (Fig. 7D). Similar to iMcm4 females, 40% of iMcm7 females died before day 8 PAE and 98% of survivors showed blocked oocyte maturation and ovarian growth (Fig. 7E), with a significantly lower length*width index in primary oocytes (Fig. 7F). After methoprene treatment, iMcm7 fat bodies showed 1.2-fold increase in mean nuclear diameter (Fig. 7C) and an 8C population (Fig. 7D), accompanied by slight but significant increase in *VgA* mRNA levels and oocyte growth (Fig. 7A, F). These observations suggest that increases in ploidy and *Vg* transcripts may be time sensitive.

## Discussion

### Mcm proteins and JH-dependent vitellogenesis

Induction of Vg synthesis by JH has been shown in the beetle *T. castaneum*, the bug *P. apterus* and the German cockroach *Blattella germanica*, as well as in *L. migratoria* [1], [11], [12], [20]. In locusts, application of JH or JHA to JH-deprived fat body induces *Vg* transcription with a lag time of 12–24 h [39], [40]. The lag can be extended by inhibition of protein synthesis with cycloheximide [40] or shortened by prior administration of JH or JHA at a sub-threshold dose [1]. Similarly, *Vg* transcription in *B. germanica* is abolished by cycloheximide treatment and accelerated by priming with a low dose of JHA [41], [42]. These observations have raised the hypothesis that this ‘late response’ of *Vg* expression by JH requires the synthesis of protein factors [1], [11], [12].

Here we show that JH induced the expression of six *Mcm* genes at 12 h post methoprene treatment (Fig. 4, Fig. S3). Of these, three *Mcm* genes were repressed by *Met* knockdown (Fig. 4, Fig. S6). Since upstream sequence was not available for *Mcm3*, we concentrated our efforts on the remaining two, *Mcm4* and *Mcm7*. Depletion of either *Mcm4* or *Mcm7* via RNAi resulted in the inhibition of *de novo* DNA synthesis, significantly lower ploidy, and a 99% reduction in *VgA* mRNA levels (Fig. 6, Fig. 7). As a consequence of the low production of yolk proteins, the primary oocytes remained immature. Intriguingly, methoprene application to *Mcm4*-depleted or *Mcm7*-depleted locusts did not restore DNA replication, polyploidy, *Vg* expression and oocyte growth to their normal levels, indicating that Mcm4 and Mcm7 are indispensable. Nevertheless, methoprene treatment on *Mcm7*-depleted adult females could partially rescue the defective phenotypes, suggesting that other functionally redundant genes, presumably genes coding for other Mcm subunits, may also be involved. The importance of other Mcm subunits in JH-dependent vitellogenesis cannot be ruled out because JH also stimulated their gene expression in synchrony with *Mcm4* and *Mcm7*. Thus, we suggest that JH acts on *Mcm* genes for the replication of fat body genome to facilitate *Vg* expression required for oocyte maturation in locusts.

Restricting consideration of JH regulation to vitellogenesis also shows variation within insects. In *T. castaneum*, JH regulates *Vg* expression through the insulin-like peptide (ILPs) signaling pathway [43]. Knockdown of *JHAMT* or *Met* reduced the expression of *ILP* genes and influenced fork head transcription factor FOXO phosphorylation and subcellular localization, eventually culminating in the binding of a DNA response element upstream of *Vg* [43]. In *P. apterus*, knockdown of either *Met* or *Taiman* blocks Vg synthesis and ovarian development [20]. However, the signaling molecules downstream of Met and Taiman in vitellogenesis and oocyte maturation of *P. apterus* are unknown.

### Regulation of *Mcm4* and *Mcm7* by the JH-receptor complex

By fluorescent confocal microscopy and flow cytometry, we demonstrated that either allatectomy by precocene treatment or *Met*-depletion via RNAi blocked *de novo* DNA synthesis and polyploidy in fat bodies, leading to considerably lower level of *Vg* mRNA and severely impaired oocyte growth (Fig. 1, Fig. 3). Since *Mcm4* and *Mcm7* were expressed in the presence of JH and Met, we postulated that Met could directly regulate *Mcm4* and *Mcm7* transcription. Cloning and sequence characterization of the upstream region of these two genes allowed the identification of E-box or E-box-like motifs (Fig. 4). We confirmed that locust Met and SRC bound to these sequences in the presence of JH, which induced *Mcm4* and *Mcm7* reporter activities (Fig. 5). The E-box motif located in the promoter region of *Mcm7* has also been found in the upstream of *Kr-h1* in the silkworm, *Bombyx mori* and the mosquito, *Ae. aegypti* and experimentally demonstrated to be recognized by the Met/SRC heterodimer in the presence of JH [7], [36]. Likewise, the E-box-like motif upstream of *Mcm4* is identical to a consensus sequence upstream of *Ae. aegypti ET* (*early trypsin*) and *RpS28* (*ribosomal protein S28*). Binding of Met to this sequence has also been experimentally defined [9], [34], [35]. It is striking that unlike the case for the more divergent ecdysone receptor [44]–[46], these observations demonstrate a remarkable conservation of both these elements and the JH receptor across more than 300 million years of insect evolution [47], [48].

Like other hormones, it appears that JH can recognize other response elements depending on the binding protein. For example, putative JH response elements have been reported for the *jhp21* gene of *L. migratoria* [49], the *JH esterase* gene of spruce budworm *Choristoneura fumiferana* [50], and several JH-responsive genes of the bee *Apis mellifera* and *D. melanogaster* [51]. These sequences are distinct from the E-box or E-box-like motifs. Additionally, their binding proteins, Tfp1, FKBP39 and Chd64, are not the members of bHLH-PAS family. Nonetheless, FKBP39 and Chd64 can interact with Met as evidenced by yeast two-hybrid and GST pull-down assays [51]. It is possible then that JH has both evolutionary conserved as well as divergent signaling. The JH-activated Met/SRC binding to the E-box and E-box-like motifs of *Mcm4* and *Mcm7* would be classified as an early hormone response. It is also likely that JH-induced *Vg* transcription requires other transcription factors. Indeed, in our luciferase reporter assays, neither Met nor SRC was able to induce *Vg* reporter activity. Investigation of other potential transcription factors, which have putative binding sites in the upstream of locust *Vg* is currently under investigation.

### Mcm4, Mcm7 and polyploidy

As mentioned, after JHA treatment, the expression of 16 genes associated with DNA replication were rapidly stimulated. Of these candidate sequences, we chose to focus on the *Mcm* genes because of their central position in eukaryotic DNA replication at both initiation and elongation. During the G1 phase of cell cycle, Mcm2-7 are transported to the origins of replication by the chromatin licensing and DNA replication factor 1 (Cdt1) to form the pre-replication complex [24]. However, although Mcm2-7 share significant sequence similarity at the ATPase active sites, the individual Mcm proteins contribute unequally to helicase activity, with the Mcm4/7 site most strongly associated with ATP-dependent DNA unwinding [24], [52]. Furthermore, Mcm4 and Mcm7 are the most evolutionarily primordial Mcm subunits [53]. Recruitment of Mcm4 and Mcm7 with Mcm6 can form a hexamer with DNA-unwinding activity [54]. Given the marked differences in these subunits, it might be predicted that *Mcm* genes are not essentially regulated by a single nuclear receptor or transcription factor(s). Indeed, our RNAi experiments showed that knockdown of *Met* resulted in the significantly reduced expression of *Mcm3*, *Mcm4* and *Mcm7*, but not *Mcm2*, *Mcm5* or *Mcm6*. The insignificant effect of *Met* RNAi on the expression of *Mcm2*, *Mcm5* or *Mcm6* suggests that other signaling molecules such as those that cross-talk with JH could be involved in the regulation of these *Mcm* genes. Recently, FOXO has been shown to mediate cell cycle arrest at the G1/S through influencing Cdt1 protein stability [55].

Reduced levels of Mcm2-7 result in declined ploidy in *Drosophila* nurse cells and follicle cells [56]. Enhanced DNA replication and ploidy have been reported in the fat body, midgut, salivary gland, wing imaginal discs, ovarian follicle cells and nurse cells of many insects [57]–[60], as well as in various cell types in plants and other animals [23], [61], [62]. Polyploidy, or endoreplication, is generated by repeated G/S cycles often in highly metabolically-active cells [23], [62]. The molting hormone, 20E, can also regulate DNA replication and polyploidy, but the mechanisms have not yet been clearly revealed [63]–[66]. During *Drosophila* metamorphosis, 20E negatively regulates polyploid cells in the larval midgut, fat body and salivary gland for tissue remodeling [61], [67]. Extended Notch signaling and down-regulated EcR results in an extra round of genomic DNA replication, whereas down-regulation of Notch and activation of EcR leads to the switch from endoreplication to specific gene amplification in *Drosophila* follicle cells [63]. Our data suggest that JH acts through Met/SRC on *Mcm4* and *Mcm7* to promote DNA replication so that fat body cells become polyploid in preparation for massive Vg synthesis. In *Drosophila*, endoreplication is driven by the oscillations of Cyclin E/Cdk2 activity [62]. Our RNA-seq profiling revealed that JH regulated 13 genes involved in cell cycle, including *Cyclin E* and *Cdk2* (Fig. 2, Table S1). It would be of interest to also investigate the role of these cycle genes in locust reproductive maturation.

**Figure 8:**
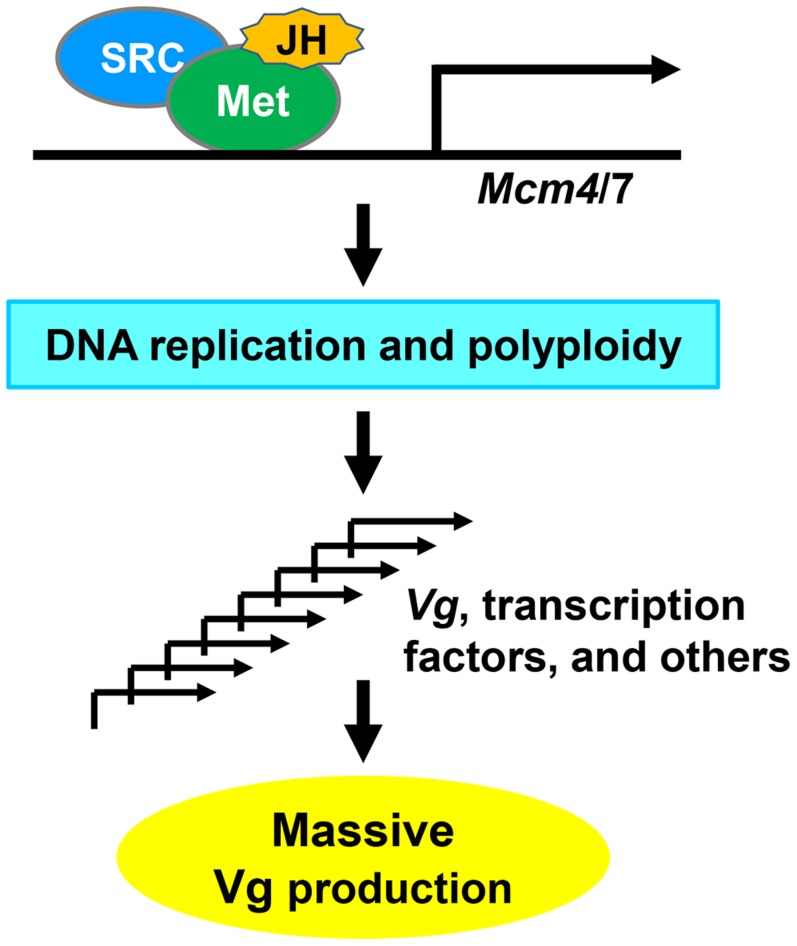
A proposed model for locust Vg synthesis regulated by the JH-receptor complex acting on *Mcm4* and *Mcm7*. An active JH-receptor complex comprised of Met and SRC in the presence of JH induces the transcription of *Mcm4* and *Mcm7* to replicate the whole genome of fat body cells. The increased ploidy leads to multiple copies of *Vg* as well as genes coding for transcription factors and other regulatory proteins that directly or indirectly regulate Vg expression. These together ensure the massive synthesis of Vg and possibly other proteins for oocyte maturation and female fertility.

Based on our results, we propose a model (Fig. 8) for locust vitellogenesis regulated by the JH-receptor complex acting on *Mcm4* and *Mcm7*. Met mediates JH action in vitellogenesis through direct regulation of *Mcm4* and *Mcm7* to replicate the fat body genome. Enhanced DNA replication and polyploidization give rise to multiple sets of chromosomes, including multiple copies of *Vg* as well as genes coding for transcription factors and other regulatory proteins that directly or indirectly regulate Vg synthesis. Together, these coordinately lead to the massive Vg production required for oocyte maturation and egg production.

## Materials and Methods

### Experimental animals

Migratory locusts were reared in the gregarious phase at a density of 200–300 locusts in each well-ventilated cage (25 cm×25 cm×25 cm) under a photoperiod of 14L:10D and at 30±2°C. They were fed with wheat bran *ad libitum* plus wheat seedlings provided once daily. JH-deprived female adult locusts were obtained by the topical application of 500 µg ethoxyprecocene (precocene III) (Sigma-Aldrich) per locust to inactivate the corpora allata within 12 h after adult eclosion. To restore JH activity, the active JH analog, s-(+)-methoprene (Santa Cruz Biotech) was topically applied to the locusts (150 µg per locust) 10 d after ethoxyprecocene treatment.

### Gene profiling and data processing

The RNA-seq approach was employed to compare gene expression profiles in JH-deprived fat bodies and those further treated with methoprene for 24 h. All cDNA libraries were sequenced in single read mode (Illumina Genome Analyzer). Raw RNA-seq reads were processed by trimming polyN read tails, discarding reads with base call quality ≤15. The remaining reads were mapped to the assembled transcripts based on previous deep sequencing transcriptome data [31]. The read mapping allowed two mismatches and was performed using the SOAP2 program [68]. Relative gene expression counts were normalized by transforming the raw data to reads per kilobases of transcripts per million mapped reads (RPKM) as described by Mortazavi *et al.* [69]. Differentially expressed genes between methoprene-exposed and precocene-treated fat bodies were identified using the DESeq package in R environments, which assumes a negative binomial distribution to determine significance [70]. Fold change ≥2 and *P*<0.05 were used to determine differentially expressed transcripts. Gene ontology enrichment was performed the BGI WEGO webserver (http://wego.genomics.org.cn/cgi-bin/wego/index.pl). Significant pathways were analyzed by the KEGG Orthology-Based Annotation System (KOBAS, http://kobas.cbi.pku.edu.cn).

### RNA isolation and qRT-PCR

Total RNA was extracted from fat bodies using Trizol reagent (Invitrogen). cDNA was reverse-transcribed from DNase-treated total RNA (2 µg) by MMLV reverse transcriptase (Promega). qRT-PCR was conducted using an MX3000P spectrofluorometric thermal cycler (Stratagene) and RealMasterMix (SYBR Green) kit (Tiangen), initiated at 95°C for 2 min, followed by 40 cycles of 95°C for 20 s, 58°C for 20 s, and 68°C for 20 s. Melting curve analysis was performed to confirm the specificity of amplification. Relative mRNA levels were normalized by β-actin, and the 2^−ΔΔCt^ method was used to analyze relative gene expression levels. Primers used for qRT-PCR are listed in Table S2.

### RNA interference

Double-stranded RNA was prepared using T7 RiboMAX Express RNAi system (Promega) following the manufacturer's instructions. Adult females were intra-abdominally injected with 18 µg dsRNA (5–6 µg/µl) within 12 h after eclosion and boosted at 5 days post eclosion. For JH rescue, RNAi-treated locusts were further topically treated with methoprene (150 µg per locust) at 6 days PAE and sacrificed up to 48 h later. For RNAi in S2 cells, *Drosophila Met* and *Taiman* dsRNA (38 nM) were transfected into S2 cells using Lipofectamine 2000 (Invitrogen) and recovered after 48 h. GFP (green fluorescent protein) dsRNA, which has no endogenous mRNA target in the migratory locust and *Drosophila*, was used as a control. Primers used in the synthesis of dsRNA are included in Table S2.

### Tissue imaging, EdU staining and confocal microscopy

Ovaries and ovarioles were photographed (Canon EOS550D camera and Leica M205C stereomicroscope, respectively). Fat bodies were fixed in 4% paraformaldehyde in PBS for 30 min, and permeabilized in 0.3% Triton X-100 in PBS for 30 min at room temperature. F-actin was stained with 0.165 µM Alexa Fluor-488 Phalloidin (Invitrogen) plus 1% BSA in PBS for 30 min. Nuclei were stained with 5 µM Hoechst 33342 (Sigma-Aldrich) in PBS for 10 min. For EdU labeling, fat bodies were incubated with 0.33 mM EdU (Invitrogen) plus 10% FBS TNM-FH modified insect medium for 90 min and processed with Click-iT EdU Imaging Kits (Invitrogen) according to the manufacturer's manuals. All fluorescein-labeled samples were imaged by ZEISS LSM 710 confocal microscopy and analyzed by ZEN2010 software (Carl Zeiss).

### FACS analysis

Brain and fat body tissues were homogenized using a Dounce homogenizer. The cells were collected by centrifugation (800×g) and fixed in 70% alcohol overnight. Fixed cells were incubated in PBS buffer containing 100 µg/ml RNaseA (Promega), 50 µg/ml propidium iodide (Sigma) and 0.2% Triton X-100 for 2 h at 4°C. After filtration with a 300 mesh cell strainer (BD Falcon), the samples were analyzed using a BD FACSCalibur Flow Cytometry System and Flowjo 7.6.1 software (BD Biosciences). Brain nuclei were used as diploid reference standards [22].

### Western blot and immunoprecipitation

Locust *Met* cDNA (nt 1–3108) and *SRC* full length cDNA were amplified by PCR, cloned into pAc5.1/Flag and pAc5.1/V5 vectors (Invitrogen) respectively, and confirmed by sequencing. The Met construct contained the functional bHLH, PAS-A, PAS-B and nuclear localization domains. *Drosophila* S2 cells were transfected with pAc5.1/Flag-Met and pAc5.1/V5-SRC using Lipofectamine 2000 (Invitrogen). After 48 h, aliquots were treated with 10 µM JH III or methoprene for 6 h. Cells were lysed in the ice-cold buffer containing 50 mM Tris pH 7.5, 150 mM NaCl, 2 mM EDTA, 1 mM DTT, 1% NP-40, 1 mM PMSF, 1 mM NaF and a protease inhibitor cocktail (Roche). After incubation for 30 min at 4°C, lysates were cleared by centrifugation at 14,000×g for 10 min, fractionated on 8% SDS-PAGE and transferred to PVDF membranes (Millipore). Western blots were performed using anti-Flag antibody or anti-V5 antibody (MBL), the corresponding HRP-conjugated secondary antibodies (CWBIO), and an enhanced chemiluminescent reagent (CWBIO). Anti-actin antibody (Abmart) was used as a loading control. For immunoprecipitation, the precleared lysates were incubated with anti-V5 antibody for 60 min at 4°C, and the immunocomplexes were captured with protein A agarose (Sigma-Aldrich), washed with lysis buffer, and eluted in Laemmli sample buffer, followed by western blotting with anti-Flag antibody.

### Luciferase reporter assay

The promoter regions of *Mcm4* (nt −933 to −37) and *Mcm7* (nt −933 to −11) were amplified by PCR, separately cloned into a pGL4.10 vector (Promega), and confirmed by sequencing. *Drosophila* S2 cells that had been previously treated with *Drosophila Met* and *Taiman* dsRNA for 48 h were seeded for 24 h at a density of 1×10^5^ cells per well in a 96-well plate (Corning), and transfected with the desired plasmids using Lipofectamine 2000 (Invitrogen). For JH or JHA treatments, 10 µM JH III or methoprene were applied 48 h post transfection and for 6 h. Luciferase activity was determined using Dual-Luciferase Reporter Assay System and a GloMax 96 Microplate Luminometer (Promega).

### Electrophoresis mobility shift assays

Locust fat body nuclear protein extracts were prepared using a NE-PER Nuclear and Cytoplasmic Extraction Reagents kit (Thermo Scientific). For *Drosophila* S2 cell nuclear protein extracts containing Flag-Met and V5-SRC, S2 cells were transfected with pAc5.1/Flag-Met^1-3108^ and pAc5.1/V5-SRC using Lipofectamine 2000 (Invitrogen), treated with 10 µM JH III for 6 h at 48 h post transfection, followed by isolation with a NE-PER Nuclear and Cytoplasmic Extraction Reagents kit (Thermo Scientific). The DNA probes (Mcm4: 5′-TTTTTGTCACGCGATTTTCA-3′; Mcm7: 5′-GCAGTGTCACGTGCTTTTGC-3′) were end-labeled with γ-^32^P-ATP by T4 DNA kinase (New England Biolabs) and purified using a Sephadex G-25 column (GE Healthcare). Nuclear protein extracts were incubated with labeled probes (∼3×10^4^ cpm) in binding buffer containing 10 mM Tris pH 7.5, 50 mM NaCl, 1 mM MgCl_2_, 1 mM DTT, 1 mM EDTA, 10% glycerol and 50 ng/µl poly(dI/dC). For competition studies, 50× molar excess of unlabeled probe was added to the binding reaction. In the supershift assays, anti-Flag (Sigma-Aldrich) or anti-V5 (Invitrogen) antibody was pre-incubated with the nuclear extracts at 4°C for 1 h before the addition of the labeled probes. Pre-incubation with IgG (Sigma-Aldrich) was used as a control. The DNA-protein complex was resolved in 5% native polyacrylamide gels and visualized using X-ray film (Kodak) for several exposure periods.

### Data analysis

Statistical analyses were performed by Student's t-test using SPSS-19.0 software (SPSS). Significant difference was considered at *P*<0.05. Values are reported as mean ± SE.

## Supporting Information

Figure S1 *Vg* expression, oocyte maturation and ovarian growth after acetone treatment. (A) *VgA* mRNA levels in the fat body of adult females treated with precocene for 10 days (P10d) and those further treated with acetone for 24 h and 48 h (PA24h and PA48h, respectively). P10d was used as the calibrator. No significant difference between acetone-treatment and precocene-treatment groups; n = 6. (B) Morphology of ovaries and ovarioles. Scale bars: ovary, 5 mm; primary oocyte, 0.5 mm. (C) Statistical analysis for length*width index of primary oocytes. n.s, no significant difference; n = 30.
(TIF)

Figure S2 Fold change in relative mRNA levels of 16 genes associated with DNA replication in the fat body of adult females treated with precocene for 10 days (P10d) and those further treated with acetone for 24 h (PA24h). P10d was used as the calibrator. No significant difference was observed; n = 4–6.
(TIF)

Figure S3 Relative expression of *Mcm2*, *Mcm3*, *Mcm5* and *Mcm6* in the fat body of adult females treated with precocene for 10 days (P10d) and those further treated with methoprene for 6, 12, 24 and 48 h (PM6h, PM12h, PM24h and PM48h, respectively). P10d was used as the calibrator. *, *P*<0.05; **, *P*<0.01 and ***, *P*<0.001 compared to P10d; n = 4–6.
(TIF)

Figure S4 Relative mRNA levels of *Mcm2-7* in the fat body of adult females treated with precocene for 10 days (P10d) and those further treated with acetone for 6, 12, 24 and 48 h (PA6h, PA12h, PA24h and PA48h, respectively). P10d was used as the calibrator. No significant difference was observed; n = 4–6.
(TIF)

Figure S5 Relative mRNA levels of *Mcm2*, *Mcm3*, *Mcm5* and *Mcm6* in the fat body of female locusts collected from 0 to10 days post adult eclosion (PAE). PAE0 (the day of adult eclosion) was used as the calibrator. *, *P*<0.05; **, *P*<0.01 and ***, *P*<0.001 compared to PAE0; n = 4–6.
(TIF)

Figure S6 Effects of *Met* RNAi (iMet) on the expression of *Mcm2*, *Mcm3*, *Mcm5* and *Mcm6*. *, *P*<0.05; **, *P*<0.01 compared to the respective dsGFP controls (iGFP); n = 10–12.
(TIF)

Figure S7 EMSA using the *Mcm4* E-box-like sequence as a probe with fat body nuclear extracts of the dsGFP control (iGFP) and *Met* RNAi (iMet). Although a single band was seen in some experiments (Fig. 4E), longer exposure times in other experiments showed three major bands; an arrow indicates the most likely specific band (see Results). FP, free probe.
(TIF)

Figure S8 Knockdown of *Drosophila Met*, *Gce* and *Taiman* in S2 cells. (A) RNAi efficiency of *Drosophila Met* (*DmMet*), *Gce* (*DmGce*) and *Taiman* (*DmTaiman*) in S2 cells treated with *Drosophila Met* and *Taiman*-specific dsRNA (dsDmMet+dsDmTaiman). FlyBase ID: DmMet, FBpp0073368; DmGce, FBpp0292296; DmTaiman, FBpp0292873. *, *P*<0.05 compared to the respective dsGFP controls; n = 4. (B) dsDmMet+dsDmTaiman treatment had no significant effect on *Flag-Met* (*Met*) or *V5-SRC* (*SRC*) expression in S2 cells. n = 4.
(TIF)

Table S1 The list of up- and down-regulated genes in JH-deprived fat bodies further treated with methoprene for 24 h in RNA-seq analysis.
(XLSX)

Table S2 Primers used for qRT-PCR and RNAi.
(DOCX)
