# Supplementary figures and images for: Juvenile Hormone-Receptor Complex Acts on Mcm4 and Mcm7 to Promote Polyploidy and Vitellogenesis in the Migratory Locust
Source: PLoS Genet. 2014 Oct 23;10(10):e1004702. doi: 10.1371/journal.pgen.1004702 (PMC4207617; doi:10.1371/journal.pgen.1004702)

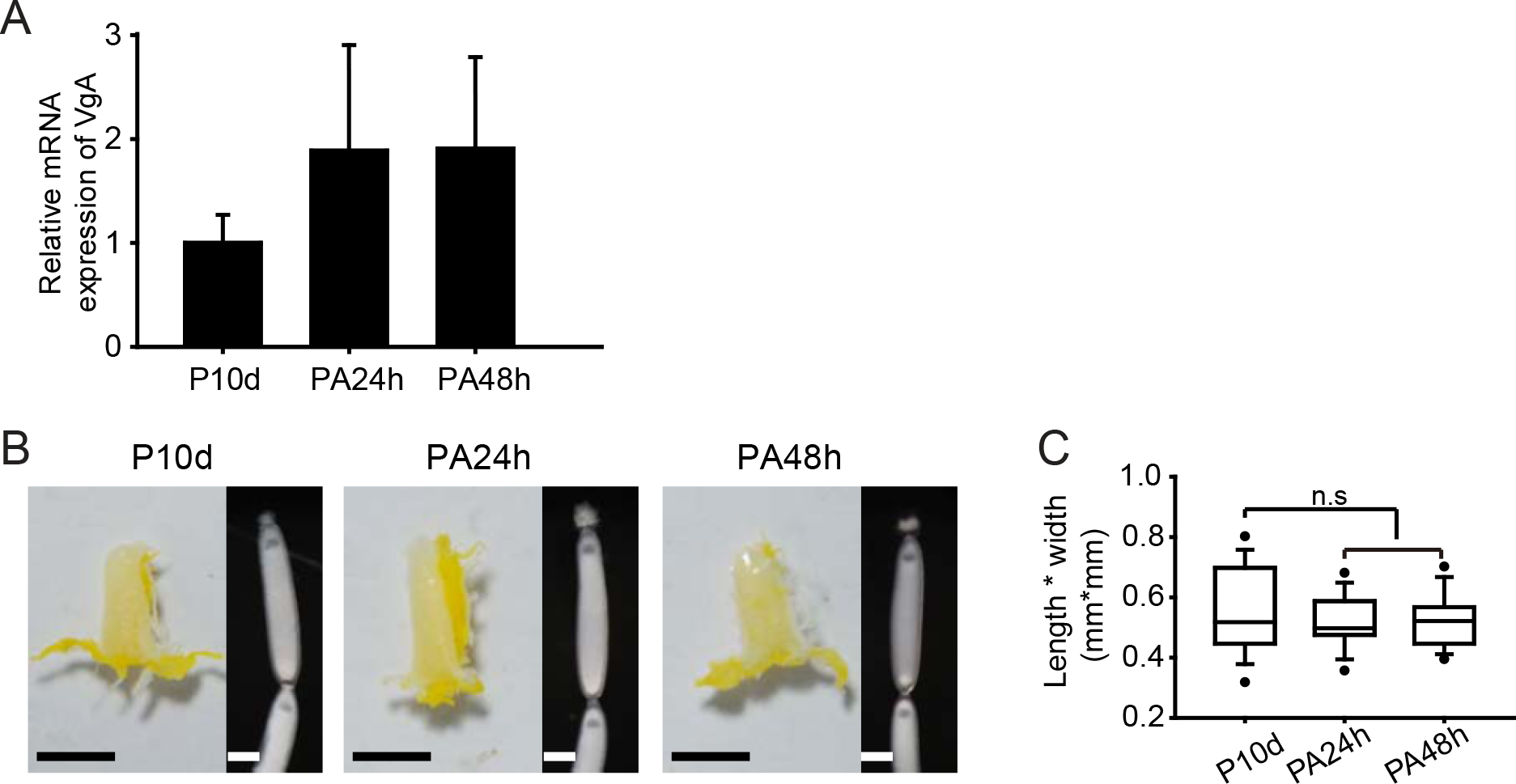

Supplement: Figure S1 — Vg expression, oocyte maturation and ovarian growth after acetone treatment. (A) VgA mRNA levels in the fat body of adult females treated with precocene for 10 days (P10d) and those further treated with acetone for 24 h and 48 h (PA24h and PA48h, respectively). P10d was used as the calibrator. No significant difference between acetone-treatment and precocene-treatment groups; n = 6. (B) Morphology of ovaries and ovarioles. Scale bars: ovary, 5 mm; primary oocyte, 0.5 mm. (C) Statistical analysis for length*width index of primary oocytes. n.s, no significant difference; n = 30. (TIF) [file pgen.1004702.s001.tif]

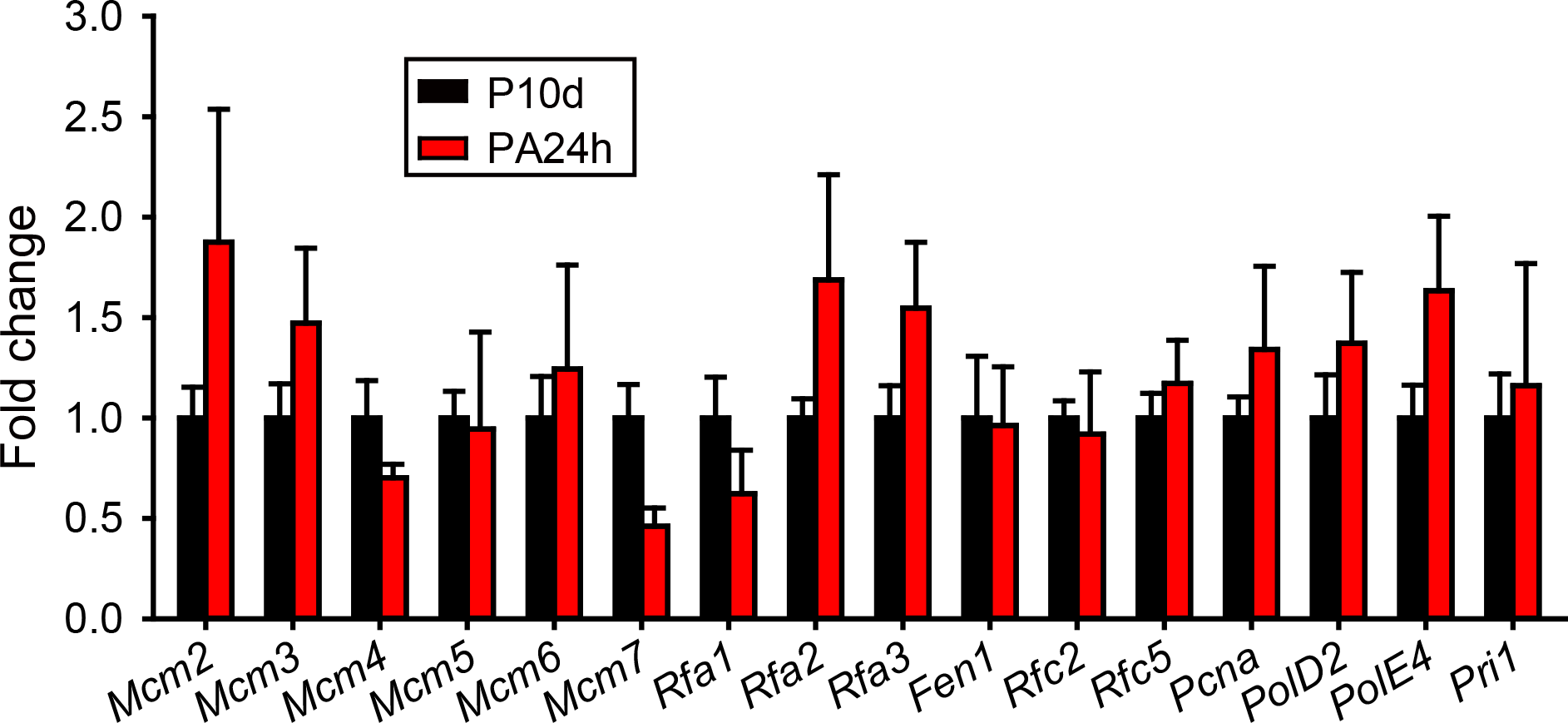

Supplement: Figure S2 — Fold change in relative mRNA levels of 16 genes associated with DNA replication in the fat body of adult females treated with precocene for 10 days (P10d) and those further treated with acetone for 24 h (PA24h). P10d was used as the calibrator. No significant difference was observed; n = 4–6. (TIF) [file pgen.1004702.s002.tif]

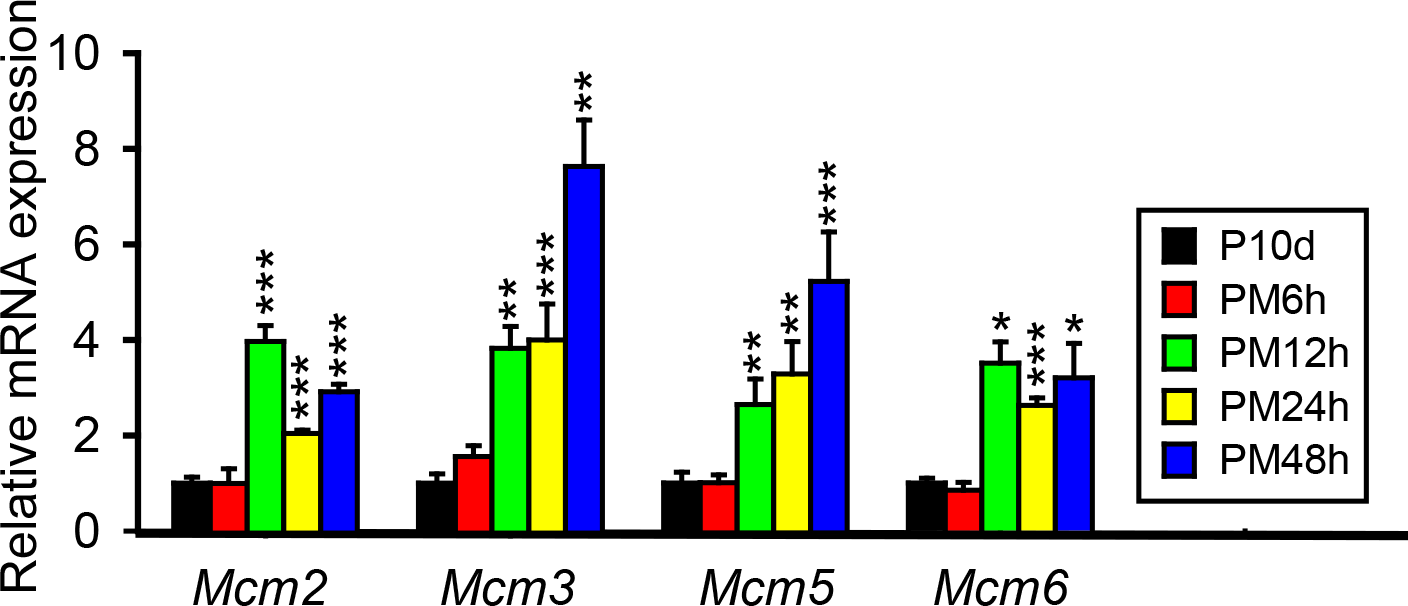

Supplement: Figure S3 — Relative expression of Mcm2, Mcm3, Mcm5 and Mcm6 in the fat body of adult females treated with precocene for 10 days (P10d) and those further treated with methoprene for 6, 12, 24 and 48 h (PM6h, PM12h, PM24h and PM48h, respectively). P10d was used as the calibrator. *, P<0.05; **, P<0.01 and ***, P<0.001 compared to P10d; n = 4–6. (TIF) [file pgen.1004702.s003.tif]

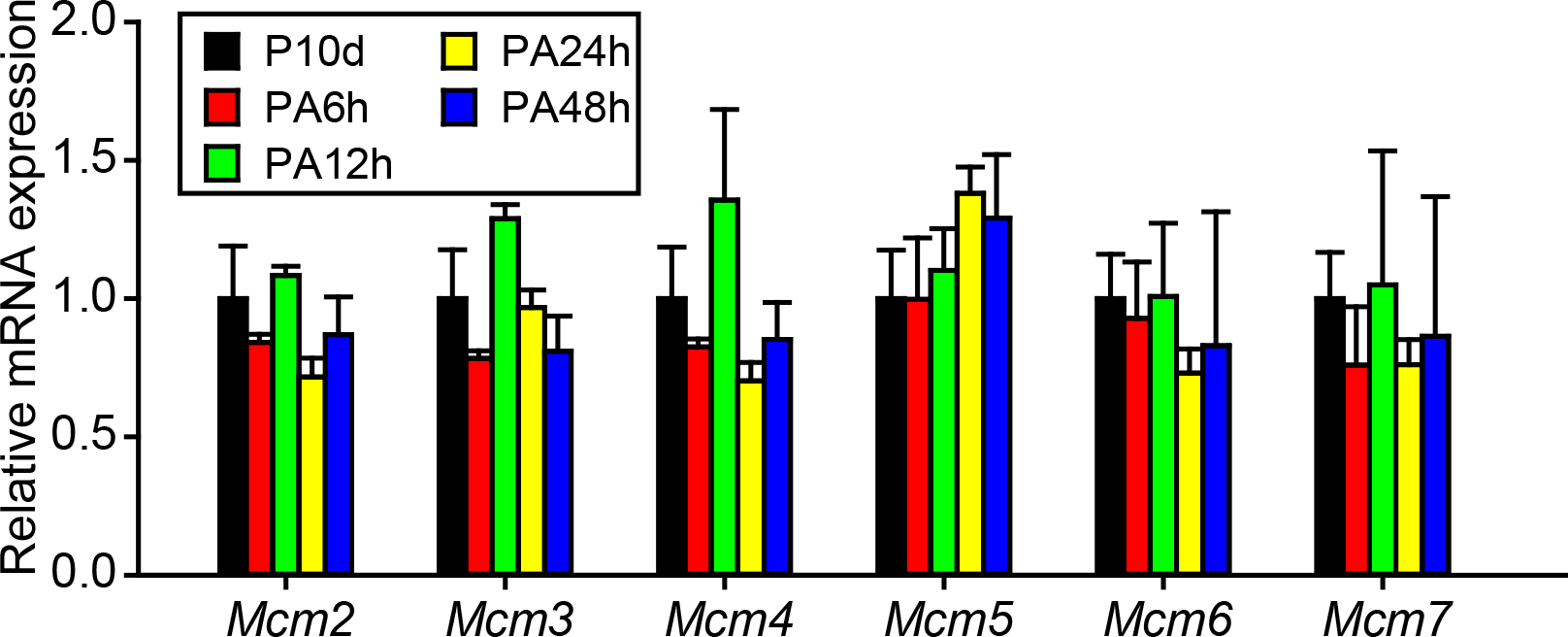

Supplement: Figure S4 — Relative mRNA levels of Mcm2-7 in the fat body of adult females treated with precocene for 10 days (P10d) and those further treated with acetone for 6, 12, 24 and 48 h (PA6h, PA12h, PA24h and PA48h, respectively). P10d was used as the calibrator. No significant difference was observed; n = 4–6. (TIF) [file pgen.1004702.s004.tif]

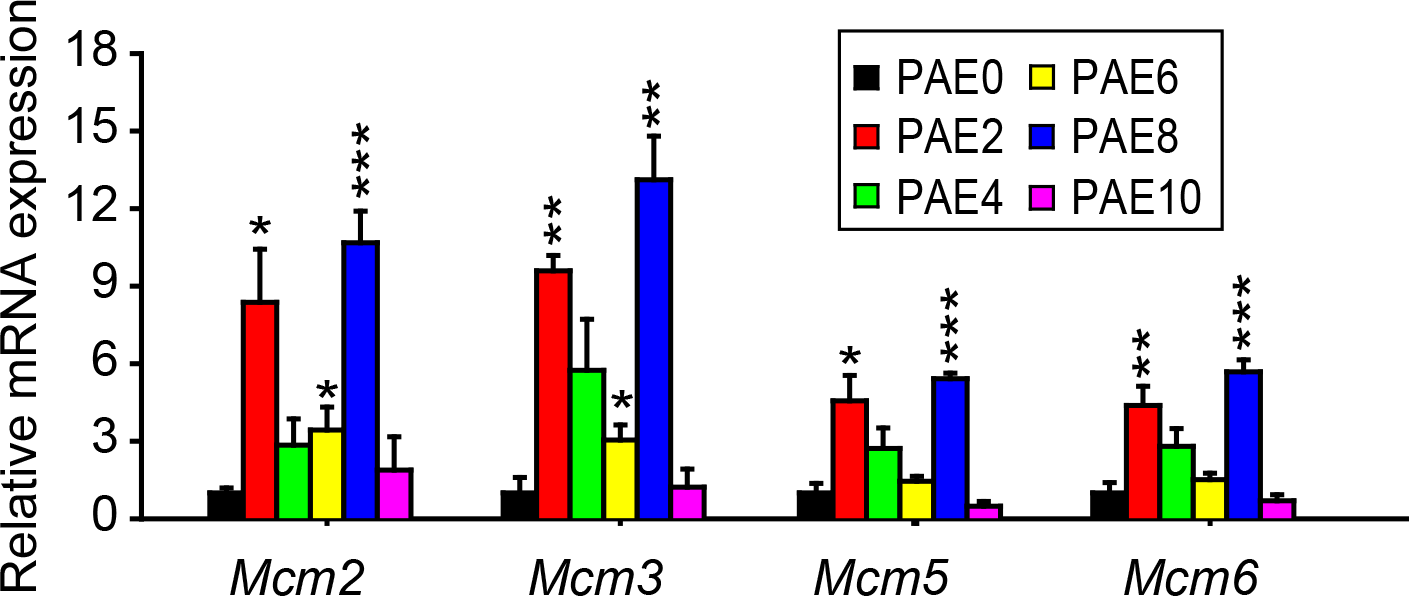

Supplement: Figure S5 — Relative mRNA levels of Mcm2, Mcm3, Mcm5 and Mcm6 in the fat body of female locusts collected from 0 to10 days post adult eclosion (PAE). PAE0 (the day of adult eclosion) was used as the calibrator. *, P<0.05; **, P<0.01 and ***, P<0.001 compared to PAE0; n = 4–6. (TIF) [file pgen.1004702.s005.tif]

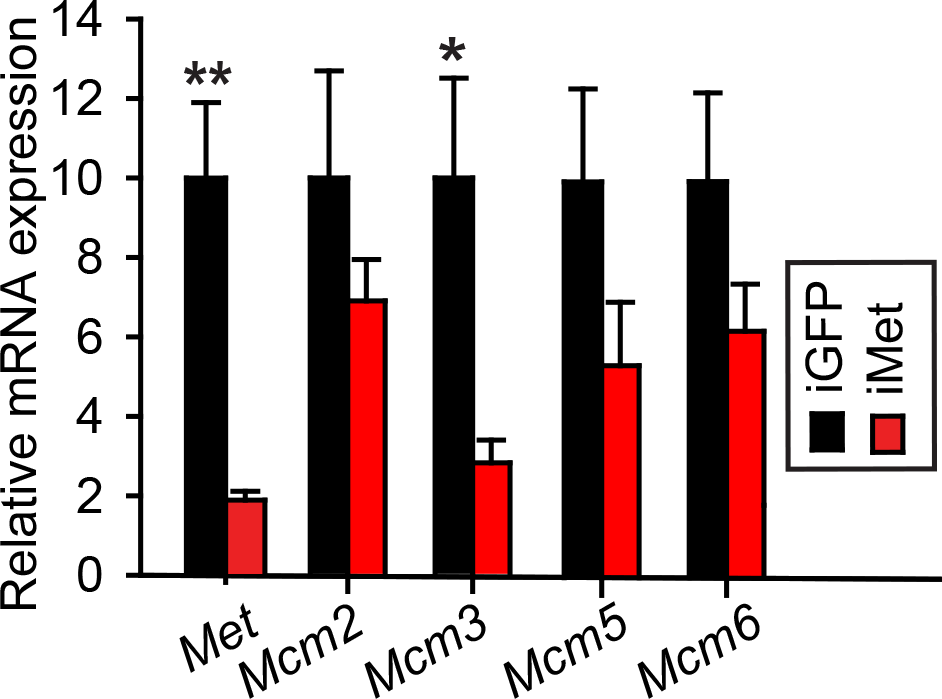

Supplement: Figure S6 — Effects of Met RNAi (iMet) on the expression of Mcm2, Mcm3, Mcm5 and Mcm6. *, P<0.05; **, P<0.01 compared to the respective dsGFP controls (iGFP); n = 10–12. (TIF) [file pgen.1004702.s006.tif]

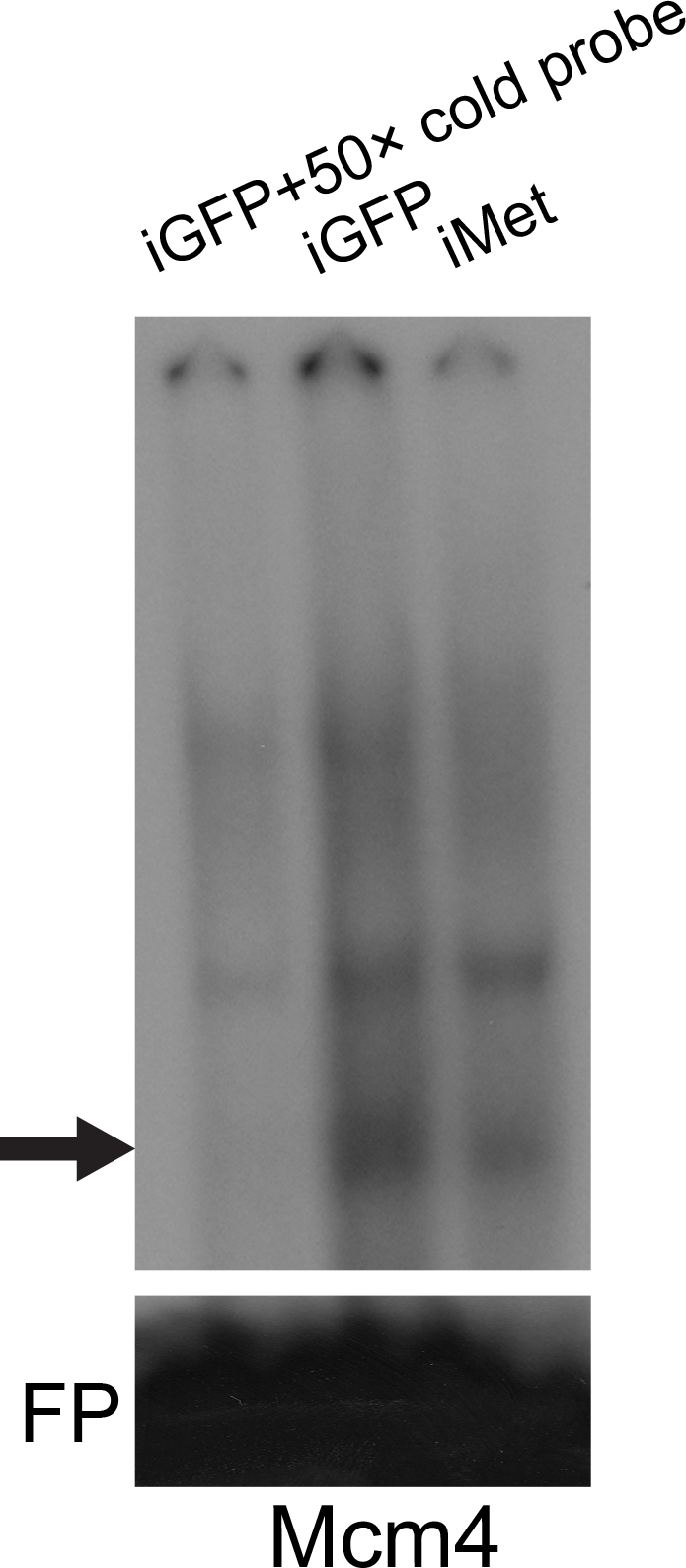

Supplement: Figure S7 — EMSA using the Mcm4 E-box-like sequence as a probe with fat body nuclear extracts of the dsGFP control (iGFP) and Met RNAi (iMet). Although a single band was seen in some experiments (Fig. 4E), longer exposure times in other experiments showed three major bands; an arrow indicates the most likely specific band (see Results). FP, free probe. (TIF) [file pgen.1004702.s007.tif]

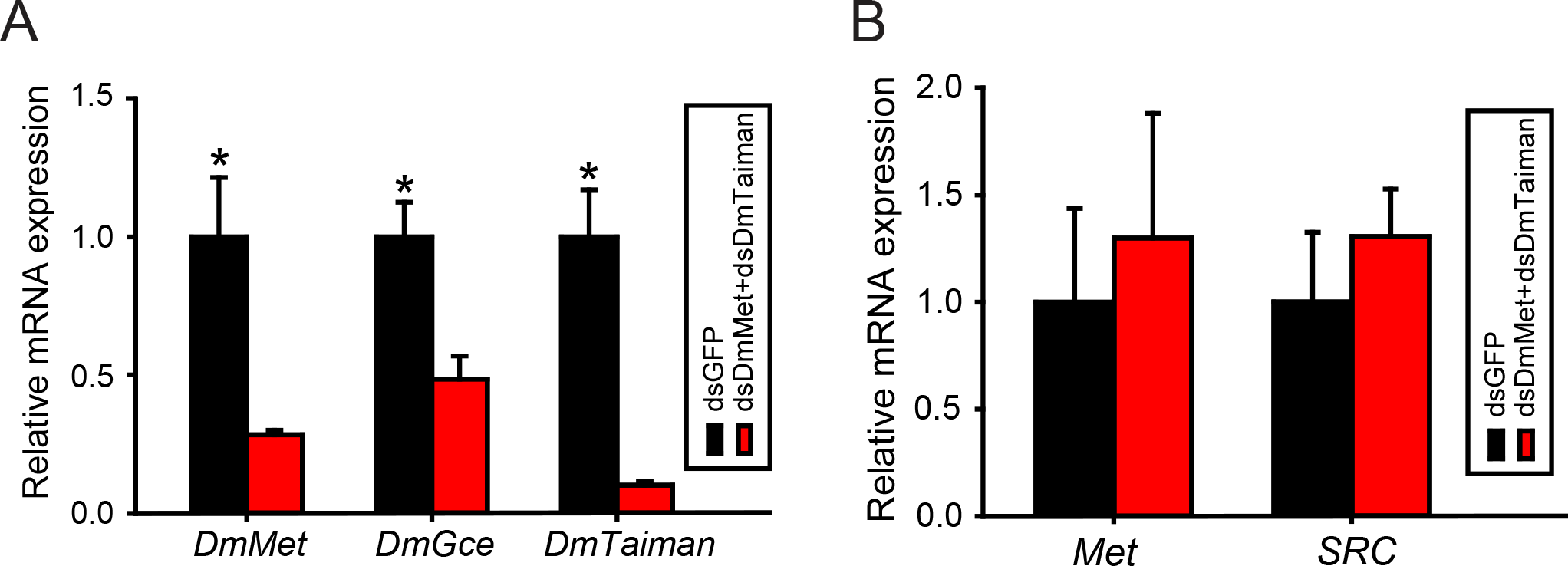

Supplement: Figure S8 — Knockdown of Drosophila Met, Gce and Taiman in S2 cells. (A) RNAi efficiency of Drosophila Met (DmMet), Gce (DmGce) and Taiman (DmTaiman) in S2 cells treated with Drosophila Met and Taiman-specific dsRNA (dsDmMet+dsDmTaiman). FlyBase ID: DmMet, FBpp0073368; DmGce, FBpp0292296; DmTaiman, FBpp0292873. *, P<0.05 compared to the respective dsGFP controls; n = 4. (B) dsDmMet+dsDmTaiman treatment had no significant effect on Flag-Met (Met) or V5-SRC (SRC) expression in S2 cells. n = 4. (TIF) [file pgen.1004702.s008.tif]
