# Supplementary material for: Juvenile Hormone-Receptor Complex Acts on Mcm4 and Mcm7 to Promote Polyploidy and Vitellogenesis in the Migratory Locust
Source: PLoS Genet. 2014 Oct 23;10(10):e1004702. doi: 10.1371/journal.pgen.1004702 (PMC4207617; doi:10.1371/journal.pgen.1004702)
Supplement: Table S2 — Primers used for qRT-PCR and RNAi. (DOCX) [file pgen.1004702.s010.docx]

**Table S2. Primers used for qRT-PCR and RNAi**

|  | **Gene** | **Forward primer** | **Reverse primer** |
| --- | --- | --- | --- |
| qRT-  PCR | *VgA* | CCCACAAGAAGCACAGAACG | TTGGTCGCCATCAACAGAAG |
|  | *Met* | GTGCCTGAAGAAGAAGAAC | GGAGGTGATGAAGGAGAG |
|  | *Mcm2* | TGGTGAACATGGAGGAGACAGT | GTGGGATACGACCAGCAGGAA |
|  | *Mcm3* | CACCACCAACTTCATCACCTAA | TTCACTGTTTGCGAACTCTGTC |
|  | *Mcm4* | GGCGAATTGCGGAACCTACC | GCAGGCATATCATCAGGCGATT |
|  | *Mcm5* | CCAAAGAGTCGTGGAAGGGAA | TAGCAGCTAAACGGCGGAAG |
|  | *Mcm6* | ATACACGGCAACGACGAAGT | CGCTGATGAACGGCTTGAAC |
|  | *Mcm7* | ACGAGTTTGACAAGATGGCTGAC | ACGACCATAGGCTGGATTTGC |
|  | *Rfa1* | GTCACAGACATCCCTCATATTACT | CCGGTTTGTTGTTCTAGCC |
|  | *Rfa2* | AGTTGAAACGACAAATGGAATG | ACCACTGACAATGTTGCTGCT |
|  | *Rfa3* | TGGAAACGACTGATGGAAGAG | AGCAGTGGGTCCCTGACAAAT |
|  | *Fen1* | TGCGACAGCAACTGAAGACA | TCACATCCCAACAAGATACAAAG |
|  | *Rfc2* | AATGAAGAAGGTGTAGCCCGC | CCCCAAGCAAAACTCTAGCAA |
|  | *Rfc5* | AGTGCAGGGCACAAACTTATT | GAGACTGGAGGGCTGGGATTA |
|  | *Pcna* | ATGCAGGAACCAGTCACCTTG | GTAGCCCATATCCGCTATTTT |
|  | *PolD2* | GCTTGGTGGTCTACTGGGAG | GTGCACTGGAGGAGGATTGT |
|  | *PolE4* | GCTGAGGAAAAGGATGGAGAA | CCCAATAGGTAAACGGACGAG |
|  | *Pri1* | AAAGACCATCGCTCAGTCACAC | GCAACACTCATAAATCGCCAACA |
|  | *DmMet* | CGTCCTTAGATTCGCCACCC | GAGGCAGACATACCCGTTCC |
|  | *DmGce* | CTCAGTCCCTTCACCTTCAT | ACCTTGTTCGTCTCCTTGTC |
|  | *DmTaiman* | AGCGATGTAAAGCCCGAGA | AAAGCAGCATTCCACCCAC |
| RNAi | *GFP* | CACAAGTTCAGCGTGTCCG | GTTCACCTTGATGCCGTTC |
|  | *Met* | TTAGGGCAGCATCAGAAAG | TCGTCGGGAGGAAGTGTAT |
|  | *Mcm4* | CGCCGTGAAAGAGGTGAAGAAG | AAGCAGGTGGAACTGACTGATGA |
|  | *Mcm7* | GGAACAGCAGACCATTACCATT | ACATCAGCCAGCCTCAGAC |
|  | *DmMet* | CTGCCAACTATCCGATTGTCTC | CTCTCGCCGTAGTCACTGTT |
|  | *DmTaiman* | AGCATCAGCACCAGCATCA | GTCGTTGTCGTAGAGTTGTTGT |
